# Use of artificial neural networks in the prognosis of musculoskeletal diseases—a scoping review

**DOI:** 10.1186/s12891-023-06195-2

**Published:** 2023-02-01

**Authors:** Fanji Qiu, Jinfeng Li, Rongrong Zhang, Kirsten Legerlotz

**Affiliations:** 1 Movement Biomechanics, Institute of Sport Sciences, Humboldt‐Universität zu Berlin, Unter Den Linden 6, 10099 Berlin, Germany; 2 Department of Kinesiology, Iowa State University, Ames, 50011 IA USA; 3 School of Control and Computer Engineering, North China Electric Power University, 102206 Beijing, China

**Keywords:** Machine learning, Musculoskeletal diseases, Prediction

## Abstract

To determine the current evidence on artificial neural network (ANN) in prognostic studies of musculoskeletal diseases (MSD) and to assess the accuracy of ANN in predicting the prognosis of patients with MSD. The scoping review was reported under the Preferred Items for Systematic Reviews and the Meta-Analyses extension for Scope Reviews (PRISMA-ScR). Cochrane Library, Embase, Pubmed, and Web of science core collection were searched from inception to January 2023. Studies were eligible if they used ANN to make predictions about MSD prognosis. Variables, model prediction accuracy, and disease type used in the ANN model were extracted and charted, then presented as a table along with narrative synthesis. Eighteen Studies were included in this scoping review, with 16 different types of musculoskeletal diseases. The accuracy of the ANN model predictions ranged from 0.542 to 0.947. ANN models were more accurate compared to traditional logistic regression models. This scoping review suggests that ANN can predict the prognosis of musculoskeletal diseases, which has the potential to be applied to different types of MSD.

**Supplementary Information:**

The online version contains supplementary material available at 10.1186/s12891-023-06195-2.

## Introduction

Artificial intelligence (AI) has emerged as an opportunity allowing numerous practical applications [1, 2]. Artificial neural network (ANN), as an important branch of modern artificial intelligence technology, have been widely used in modern medicine due to their strong learning capability and stable feature recognition and prediction of functions [3, 4]. ANN is an information processing system established by imitating the structure and function of the neural network of the brain. Dr. Robert H. Nielsen, the inventor of the neural computer, defines a neural network as a computational system consisting of many simple, highly interconnected processing elements that can handle real-world problems by dynamically reacting to external inputs [5]. ANN can extract feature information from existing clinical experience and massive external input data and then perform self-learning, so ANN does not require a detailed description of the disease, but only basic information about the patients to obtain the corresponding diagnosis and treatment plan [6].

The application of ANN may be particularly useful in areas such as MSD as various clinical indicators related to musculoskeletal diseases are suitable for processing in ANN. Since ANN is able to use a large amount of those clinical data for machine learning, it may generate a stable clinical prediction model. In addition, musculoskeletal diseases (MSD) are associated with high morbidity and mortality and also lead to high healthcare costs [7, 8]. Globally, MSDs account for 21% of total morbidity and affect more than 25% of the population [9]. In the United States, approximately 130 million health care visits and approximately 70 million physician visits are associated with MSD each year [10], and MSD patients account for more than 25% of emergency department visits [11]. MSD is also the second most common cause of disability worldwide, with an estimated 45% increase in disability due to MSD disease, particularly osteoarthritis (OA), from 1990 to 2010, and the number of people suffering from MSD is expected to continue to increase with the impact of obesity, sedentary immobility, and an aging population [12–14].

Given the high prevalence and variety of MSDs (from tendon injuries in young athletes to degenerative diseases in the elderly), and the fact that some disease types are chronic or even incurable, finding a method that can effectively determine prognosis can help MSD patients better manage their disease and alleviate the burden of disease [15, 16], and may also reduce healthcare costs.

There is emerging evidence, that ANN can be applied to predict injury rates or treatment outcome in MSD. In postmenopausal women, An ANN model was used to predict fragility fractures using the bone strain index (BSI) and dual-energy x-ray absorptiometry (DXA), achieving a prediction accuracy of 79.56% [17]. In patients with chronic plantar fasciitis (CPF), ANN was used to determine the predictive factors for minimum clinically successful therapy (MCST) after extracorporeal shockwave treatment and found that the overall accuracy of the predictive model was 92.5% [18]. In patients with lumbar disc herniation (LDH), ANN model could predict the efficiency of hospitalization satisfaction with an accuracy of 96% [19]. Prognosis studies aim to predict the likelihood of disease progression related to different events (e.g., bone fracture, conjunctivitis) or explore the factors influencing disease outcomes [20, 21]. The use of ANN in prognosis may help doctors and patients to better understand the status and progression of the patient’s disease, resulting in individualized and more appropriate clinical decisions, which may reduce medical costs and improve recovery outcomes.

ANN has the potential to predict the prognosis of MSD by various variables such as patient's age, gender, treatment modality and disease severity. Therefore, the aims of this scoping review were threefold: (1) compile articles on the prognostic application of ANN in MSD, (2) investigate the accuracy of ANN in predicting the prognosis of MSD, (3) whether ANN has better predictive ability than other models.

## Methods

This scoping review complies with all of the Preferred Items for Systematic Reviews and the Meta-Analyses extension for Scope Reviews (PRISMA-SCR) guidelines [22] and reports the required information accordingly. In addition, we also implemented the stages set out by Arksey and O'Malley [23] in the current scoping review. The protocol for this scoping review (https://doi.org/10.17605/OSF.IO/7UGFV) was registered at the Open Science Framework Registries (OSF).

### Identifying the research question

This scoping review examined peer-reviewed articles on the use of ANN for prognosis prediction in MSD. Our scoping review identified the following questions: 1. Can ANN use basic clinical information of patients with musculoskeletal disorders to predict the prognosis of patients? 2. How effective and accurate was the ANN model used in prognosis studies? 3. Was ANN more effective than other machine learning methods or logistic regression? 4. What metrics were used in the included studies to predict prognostic outcomes?

### Identifying relevant studies

Inclusion and exclusion criteria were determined on the basis of our study objectives.

### Inclusion criteria

Studies applying ANN to predict the prognosis of musculoskeletal diseases, written in English.

### Exclusion criteria

Studies not related to musculoskeletal diseases, not written in English, duplicate publications, unpublished studies, literature review papers, letters to the editor, conference abstracts and animal model studies.

### Study selection

To ensure an extensive search for the inclusion of relevant articles, we searched the Cochrane library, Embase, Pubmed and the Web of science core collection. The retrieval time range was from the establishment of the database to January 7^th^, 2023. We use medical subject headings (MeSH) to facilitate literature retrieval, with three main subject headings: artificial neural networks; musculoskeletal diseases; prognosis. We applied different search strategies in different databases, and the full documentation of the final search strategy is available in the supplementation file. The database search results were imported into Endnote X9 (Thomson Reuters, NY, USA) and duplicates were removed. In order to include as much relevant literature as possible, we first performed Mesh terms searches with artificial neural networks, musculoskeletal diseases, and prognosis combined strategy, and then performed keywords researches. Titles and abstracts of retrieved articles were independently read and reviewed by FJQ and JFL, and any disagreement during the screening process was resolved through discussion and consensus with the third reviewer (RRZ). After the full text was obtained, the data was extracted from the paper.

### Charting the data

Microsoft Excel (Version 2019) was used for the extraction of study data. Data charting was performed according to our proposed questions and the information extracted included (1) basic information about the study (authors, region, year of publication, sample size, study purpose, study design), (2) characteristic information of patients (age, disease type) and (3) ANN effect evaluation method, accuracy, and platform for modeling.

### Collating, summarizing, and reporting the results

**Fig. 1:**
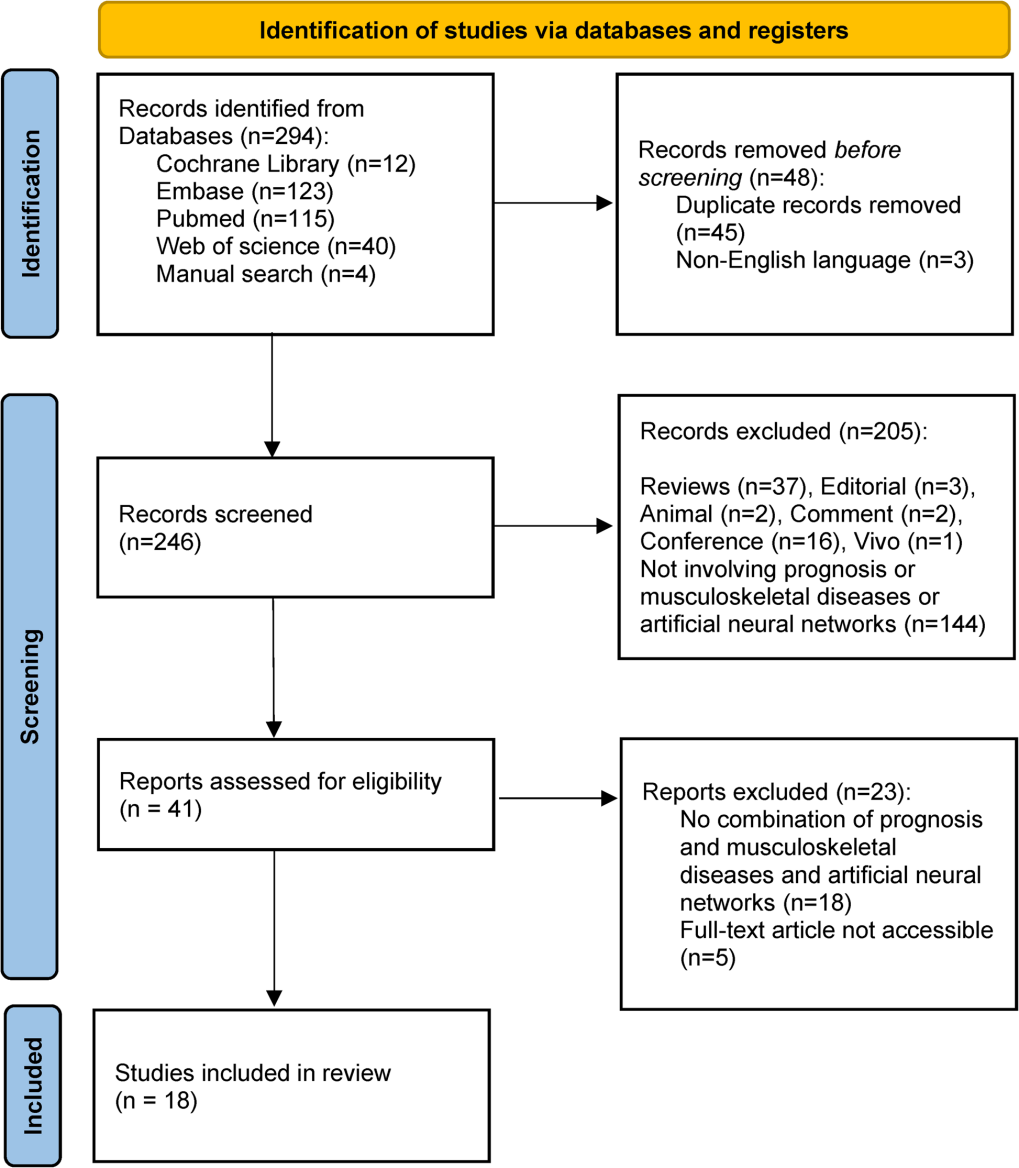
Study selection process (according to the PRISMA-ScR guidelines [22])

A total of 294 records were retrieved from the four databases, leaving 246 articles after removing duplicates and non-English studies. After reviewing the titles and abstracts according to inclusion and exclusion criteria, 205 articles were excluded. After reading the full text, 23 articles were excluded. The flowchart of the article retrieval and screening is shown in Fig. 1. We did not perform a quality assessment due to inconsistencies in the types of studies included in our study.

## Results

Eighteen papers were finally included in the systematic analysis (Table 1).

**Table 1:** Basic information of included studies

Author (region)	Year of publication	Study design	Journal	Objectives	Type of disease/injury
Alfieri et al. (USA)	2015	Cohort study	Clinical Orthopaedics and Related Research	Estimating the likelihood of wound-specific HO formation and determining (1) which model is most accurate; and (2) which technique is best suited for clinical use	Heterotopic Ossification
Almhdie et al. (France)	2022	Cohort study	Arthritis Research and Therapy	To evaluate the predictive ability of a combined approach using both TBT descriptors deep learning-based Siamese CNN tools to predict KOA progression	Knee osteoarthritis
Bevevino et al. (USA)	2014	Cross-sectional study	Clinical Orthopaedics and Related Research	Determining which model (artificial neural network and a logistic regression model) more accurately estimated the likelihood of amputation and which was better suited for clinical use	Combat-related Open Calcaneus Fractures
Bowman et al. (UK)	2018	Cohort study	Muscle and Nerve	Develop and validate a comprehensive, multivariate prognostic model for carpal tunnel surgery in a large sample of ordinary NHS surgical procedures	Carpal tunnel syndrome
Chen et al. (China)	2020	Cohort study	Medicina	To validate the accuracy of an ANN model for predicting the mortality after hip fracture surgery during the study period, and to compare performance between the ANN and Cox regression model	Hip fracture
Eller-Vainicher et al. (Italy)	2011	Cohort study	PLoS One	To Comparing ANNs and LR in recognising, on the basis of osteoporotic risk-factors and other clinical information, patients with SDI ≥ 1 and SDI ≥ 5 from those with SDI = 0	Osteoporotic fractures
Jalali et al. (USA)	2020	Cohort study	Anesthesia and analgesia	Developing a machine-learning model to predict blood product transfusion requirements for individual pediatric patients undergoing craniofacial surgery	Craniosynostosis
Kim et al. (Korea)	2022	Cross-sectional study	Pain Physician	Developed and investigated the accuracy of a CNN model for predicting therapeutic outcomes after TFESI for controlling chronic lumbosacral radicular pain	Chronic Lumbosacral Radicular Pain
Kim et al. (USA)	2018	Cohort study	Spine Deformity	To train and validate machine learning models to identify risk factors for complications following surgery for adult ASD	Adult spinal deformity
Lu et al. (USA)	2021	Cohort study	Orthopaedic journal of sports medicine	To develop and internally validate a machine-learning model to predict the outcomes after ASI	Anterior Shoulder Instability
Miyoshi et al. (Japan)	2016	Cohort study	Modern rheumatology	Develop ANN model for predicting the clinical response to IFX in RA patients	Rheumatoid Arthritis
Norgeot et al. (USA)	2019	Cohort study	JAMA Netw Open	To assess the ability of an artificial intelligence system to prognosticate the state of disease activity of patients with RA at their next clinical visit	Rheumatoid Arthritis
Salgueiro et al. (Spain)	2013	Cohort study	Pain medicine	Evaluate the ability of ANNs to predict the response of persons with FMS to a standard, 4-week interdisciplinary pain program	Fibromyalgia syndrome
Scheer et al. (USA)	2017	Cohort Study	Journal of neurosurgery. Spine	To develop a model based demographic, radiographic, and surgical factors that can predict if patients will sustain an intra/perioperative major complication	Spinal deformity
Shin et al. (Korea)	2022	Cohort study	Medicina	To investigate the usefulness of DNN models based on 18F- FDG-PET and blood inflammatory markers to assess the therapeutic response in PVO	Pyogenic vertebral osteomyelitis
Su et al. (China)	2022	Cohort study	Computational and Mathematical Methods in Medicine	To explore the prognostic factors of endoscopic surgery for OA and to predict the long-term efficacy of this type of surgery for OA by ANNs	Osteoarthritis
Wang et al. (China)	2017	Cross-sectional study	PeerJ	Using clinical examination indicators predicts interstitial lung disease among patients with rheumatoid arthritis	Rheumatoid arthritis
Yahara et al. (Japan)	2022	Cohort study	BMC musculoskeletal disorders	To develop a new diagnostic platform using DCNN to predict the risk of scoliosis progression in patients with AIS	Adolescent idiopathic scoliosis

*AIS* Adolescent idiopathic scoliosis, *ASD* Adult spinal deformity, *DNN* Deep neural network, *DCNN* Deep convolutional neural network, *FDG-PET* Fluorodeoxyglucose positron emission tomography, *FMS* Fibromyalgia syndrome, *IFX* Infliximab, *KOA* Knee osteoarthritis, *NHS* National Health Service, *OA* Osteoarthritis, *PVO* Pyogenic vertebral osteomyelitis, *RA* Rheumatoid arthritis, *ROC* Receiver operating characteristic, *SDI* Spinal deformity index, *TBT* Trabecular bone texture

### Characteristics of included studies

The included studies were from sixteen journals, with only 2 articles each were published in Clinical Orthopaedics and Related Research [24, 25] and Medicina [26, 27]. Thirty-three percent of these studies (7/18) were conducted in the United States [24, 25, 28–32], with 17% (3) in China [26, 33, 34]. Study designs included cohort study (83%; 15) and cross-sectional database study (17%; 3). The included studies investigated 16 different musculoskeletal diseases. The detailed information of study characteristics was shown in Table 2.

**Table 2:** Demographics of subjects

Study	Sample size (n)	Age (Years^a^)	Sex
Alfieri et al	72	22.0 (21.0, 26.0)	m
Alfieri et al	2571	-	-
Bevevino et al	134	24.0 (22.0, 28.0)	130 m / 4f
Bowman et al	885	66.0 (21.0–93.0) ^m^, 62.0 (20.0–100.0) ^f^	306 m / 579f
Chen et al	10,534	68.3 (14.6)	4469 m / 6065f
Eller-Vainicher et al	372	68.0 (8.5)	f
Jalali et al	2143	-	-
Kim et al. (Korea)	503	59.2 (14.4)	226 m / 277f
Kim et al. (USA)	5794	59.5	2376 m / 3418f
Lu et al	654	21.7 (17.0, 29.0)	500 m / 154f
Miyoshi et al	179	-	36 m / 143f
Norgeot et al	820	57.0(15.0)-60.0(15.0)	148 m / 672f
Salgueiro et al	72	41.50 (21.0–59.0)	3 m / 69f
Scheer et al	557	57.5 (15.3)	118 m / 439f
Shin et al	74	67.27 (11.18)	47 m / 27f
Su et al	236	67 (60–70)	90 m / 146
Wang et al	838	53.4 (12.5)	-
Yahara et al	58	12.5 (1.4)	9 m / 49f

*f* Female, *m* Male

^a^ Age was presented as Mean, Mean (SD) and Mean (Range)

### Characteristics of participants

The mean age of the patients ranged from 12.5 to 100.0 years [35, 36] (Table 2). Two of the studies included exclusively female or male participants [24, 37]. The sample size ranged from 58 to 10534 [26, 36]. Two studies [28, 38] did not provide data on the age and sex of participants.

### Effects of ANN in prognosis

The areas under the curve (AUC) served as a metric to evaluate the accuracy of ANN. The overall accuracy ranged from 0.542 to 0.947 (Table 3). 80.0% (8/10) of the studies showed that ANN had a better prediction accuracy than logistic regression (LR) or other prediction models. Eight studies did not compare ANN with other models, and two study found that ANN model had lower prediction accuracy than gradient boosting machine (GBM) and extreme gradient boosted machine (XGBoost). MATLAB was the most frequently (3 times) used platform in ANN modeling.

**Table 3:** Characteristics of studies in terms of ANN modeling and accuracy

Author	Variables used for ANN model	Method to evaluate accuracy	Comparing with other method	Results of ANN accuracy	Platform for ANN modeling
Alfieri et al	The gene transcript expression of 190 wound healing, inflammatory, osteogenic, and vascular genes	ROC curve	Better than LASSO LR	AUC = 0.780	Oncogenomics Online ANN Analysis system
Almhdie et al	KL grades, OARSI grades, demographic, WOMAC pain, race, and history of knee injury, and TBT	ROC curve	Better than LR	AUC^OAI^ = 0.75; AUC^MOST^ = 0.81	ImageNet
Bevevino et al	Demographics, mechanism of injury, wound size and location, fracture types, interval and definitive treatment procedures, rotational or free tissue transfer, skin graft, and neurovascular procedures, and ipsilateral and contralateral orthopaedic injuries	ROC curve	Better than LR and random forest model	AUC = 0.800 (95% CI: 0.770, 0.820)	SAS
Bowman et al	Demographic, clinical and neurophysiological variables^a^	ROC curve	Better than LR	AUC = 0.767	MATLAB
Chen et al	Demographic, referral to lower-level medical institutions, urbanization, socioeconomic status, number of comorbidities, intracapsular fracture, hospital level, hospital volume, and surgeon volume	ROC curve	Better than Cox regression model	AUC = 0.930 (95% CI: 0.900, 0.960)	STATISTICA
Eller-Vainicher et al	Menopausal age, number of pregnancies, breast feeding, smoking habits, alcohol consumption, previous clinical fragility fractures at spine, ribs, wrist and hip, BMI, calcium intake, co-morbidities^b^	ROC curve	Better than LR	AUC^SDI≥1^ = 0.714; AUC^SDI≥5^ = 0.823	TWIST system
Jalali et al	Demographics, intra/postoperative transfusion, intraoperative surgical and anesthetic management, postoperative management and laboratory results, the occurrence of prespecified intraoperative and postoperative complications, and the length of intensive care unit and hospital stay in calendar days	ROC curve	Inferior than GBM	AUC = 0.790;	Python
Kim et al. (Korea)	T2-sagittal consecutive lumbar spine MR images, T2-weighted sagittal lumbar spine MR slices, MR images obtained prior to the TFESI	ROC curve	-	AUC = 0.827(95% CI, 0.774,0.909)	Python
Kim et al. (USA)	Demographic, diabetes, smoking, steroid use, coagulopathy, functional status, ASA class O3, BMI, pulmonary comorbidities, and cardiac comorbidities	ROC curve	Better than LR	AUC^cardiac^ = 0.768; AUC^VTE^ = 0.542; AUC^wound^ = 0.606; AUC^mortality^ = 0.844	MATLAB
Lu et al	Age, sex, body mass index, type of sports participation, clinically documented ligamentous laxity, clinical history of instability, radiographic findings, management, recurrent instability, and development of clinically symptomatic osteoarthritis	ROC curve	Inferior than XGBoost	AUC^recurrence^ = 0.823 (95% CI, 0.821,0.824); AUC^surgery^ = 0.689 (95% CI, 0.687,0.692); AUC^osteoarthritis^ = 0.692 (95% CI, 0.687,0.697)	R
Miyoshi et al	Demographic, ESR, TEN, ALB, MONO, RBC, PSL, MTX, HbA1c and Pre bio	ROC curve	-	AUC = 0.750	WEKA software package
Norgeot et al	Prior CDAI score, ESR and CRP level, DMARDs, oral and injected glucocorticoids, autoantibodies, age, sex, and race/ethnicity	ROC curve	-	AUC^UH^ = 0.910 (95% CI, 0.860,0.960); AUC^SNH^ = 0.740 (95% CI, 0.650,0.830)	Github
Salgueiro et al	MPQ, the HAQ-DI, and the anxiety subscale of HADS	ROC curve	Better than LR	AUC^ANN1^ = 0.917; AUC^ANN2^ = 0.947	-
Scheer et al	Demographic, radiographic, and surgical factors	ROC curve	-	AUC = 0.890	SPSS Modeler
Shin et al	Changes in clinical symptoms and blood inflammatory markers	ROC curve	-	AUC = 0.902 (95%CI, 0.804, 0.999)	Keras and TensorFlow
Su et al	Sex, age, BMI, region, morning stiffness time, step count, and osteophyte area	ROC curve	-	AUC^worse^ = 0.814; AUC^unchanged^ = 0.700; AUC^improved^ = 0.761	R
Wang et al	Age, EO, PLT, WBC, NEUT, U-SG, U-WBC, U-WBC	ROC curve	-	AUC^ILD^ = 0.792; AUC^PF^ = 0.751	STATISTICA
Yahara et al	Frontal view of the total spine radiographs: the C7 vertebra and diaphragm; diaphragm and ilium; and C7 vertebra and ilium	ROC curve	-	AUC = 0.700	MATLAB

*Ada* Adaptive boosting, *AIS* Adolescent idiopathic scoliosis, *ASA* American Societies of Anesthesiologists, *AUC* Areas under curve, *BMI* Body mass index, *CDAI* Clinical disease activity index, *COPD* Chronic obstructive pulmonary disease, *CRP* C-reactive protein B33, *DAS* Disease activity score, *DMARD* Disease-modifying antirheumatic drug, *EN* Elastic net, *EO* Eosinophil count, *ESR* Erythrocyte sedimentatio + B4n rate, *GBM* Gradient boosting machine, *HADS* Hospital anxiety and depression scale, *HAQ* Health assessment questionnaire, *ILD* Interstitial lung disease, *LR* Logistic regression, *MONO* Monocytes, *MPQ* McGill pain questionnaire, *MR* Magnetic resonance, *MTX* Methotrexate, *NEUT* Neutrophil count, *NN* Neural network, *PLT* Blood platelet count, *PSL* Prednisolone, *Pre bio* Previous use of biologic agents before infliximab, *RBC* Red blood cells, *ROC* Receiver operating curve, *RF* Random forest, *SDI* Spinal deformity index, *SNH* Safety-net hospital, *SVM* Support vector machine, *TFESI* Transforaminal epidural steroid injections, *TEN* 28 tender joint count, *WBC* White blood cell count, *UH* University hospital, *U-SG* Urine specific gravity, *U-WBC* White blood cell count in urine, *U-WBCH* White blood cell (high power field) in urine, *VTE* Venous thromboembolism, *XGBoost* Extreme gradient boosted machine.3 m = 3 months; 12 m = 12 months

^a^ i.e. CTS: carpal tunnel syndrome, FSS – functional status score, NCS – nerve conduction studies, SSS – symptom severity score

^b^ i.e. arterial hypertension, dyslipidemia, gastric/esophagus disease, anxiety, depression, COPD, osteoarthritis, kidney stones, type 2 diabetes mellitus

## Discussion

In the treatment and rehabilitation of musculoskeletal diseases, the consideration of different symptoms and demographic data to accurately predict clinical outcomes can aid in the clinical decision-making process to provide effective and adequate treatment for patients. The main findings of this scoping review were that in different types of musculoskeletal diseases, ANN can provide accurate predictions regarding the prognosis of patients and more accurate compared to other models.

In the prognostic studies of musculoskeletal diseases, ANN was able to make accurate predictions using demographic characteristics and patient clinical characteristics as the main parameters (features). Using bone mineral density and the bone strain index as parameters, ANN predicted the occurrence of vertebral fractures (VF) in postmenopausal women in 79.56% of cases [17]. When trabeculae microstructure parameters were used as the main variable, ANN models (AUC = 0.928) were more accurate than LR and random forest (RF) in predicting marginal bone loss [39]. Using disease history and lifestyle habits of 1419 patients as parameters to predict the risk of osteoporosis in adults, the ANN model was able to accurately predict the risk of osteoporosis (AUC = 0.901), outperforming the predictive power of Deep Belief Network (DBN), Support Vector Machine (SVM) and combinatorial heuristic method (Genetic Algorithm—Decision Tree) [40]. It is encouraging that in different types of MSD, ANNs are more accurate and outperform other prediction models in disease risk prediction.

ANN can also be applied in the area of predicting rehabilitation decisions and rehabilitation outcomes, with predictions being accurate. In a study using the demographic and clinical characteristics of 170 patients to predict rehabilitation options for patients with osteoarthritis of the knee, the developed medical decision support system was able to accurately predict treatment options for 87% of patients, thus effectively assisting clinical rehabilitation staff to develop OA rehabilitation plans [41]. A study using ANN to predict patient function one year after spinal cord injury found that ANN were highly accurate in predicting walking status (AUC range between 0.86 and 0.90) and moderately accurate when used to predict non-walking outcomes (AUC between 0.70 and 0.82), and that models generated by artificial neural networks performed better than LR [42]. The application of ANN in the rehabilitation can simplify the cumbersome manual assessment process and allow for accurate prediction of some parameters that are difficult to assess quantitatively in the clinic, saving clinical diagnosis and treatment time and reducing the workload of rehabilitation physicians and therapists.

The accuracy of prognostic prediction using ANN models varied among diseases. In patients undergoing elective adult spinal deformity procedures [43], the accuracy of ANN in predicting venous thromboembolism (VTE) and wound incidence can be considered as poor and failed (AUC^VTE^ = 0.542; AUC^wound^ = 0.606) according to generally accepted AUC accuracy classification practice [44, 45]. Compared to the other included studies, in which the ANN model prediction accuracy was higher than 0.7, a relatively low number of features was used in the ANN model. While in this study [43] 8 features were used to predict 4 different symptoms, other studies used 10 [24] to 25 [37] features to predict one symptom. A possible reason for the discrepancy in accuracy may thus be that insufficient relevant features were included in ANN models.

ANN has higher accuracy compared to traditional logistic regression models. Research in the field of bioengineering has demonstrated that ANN are superior to traditional statistical models in terms of their ability to analyze nonlinear relationships, their ability to handle relevant independent variables, and their classification accuracy [46]. The advantages of ANN are mainly in the following three aspects:

- 1. Multi-layer network structure: ANN individual neurons cooperate with each other and form a network synergy when processing information, maintaining their independence while sharing and cascading the output results with other neurons, and making the results more reliable through the use of multiple hidden layers [47, 48];
- 2. Adaptive: According to the characteristics of the information in the input neural network, ANN can continuously establish new structures consistent with external changes through learning, extracting, and collecting information required for specific tasks from the data, and summarize the acquired knowledge, thereby improving the ability of data processing [49];
- 3. Accommodating data deficiencies: In contrast to traditional models that require data completeness, artificial neural networks can maintain the validity of the model even when the patient's data is incomplete [50–52].

## Limitations

Only 56% (10/18) of the studies included in this scoping review compared the accuracy between ANN and other models, which may have limited the judgment of the effectiveness of ANN applications. This study also found that although ANN has shown excellent accuracy in its application, applying it to construct predictive models may be problematic and cause over-fitting. As a consequence, the results in the receiver operating curve may be better than actual, as patients are highly selected for inclusion. Therefore, the ANN model still needs to be externally validated after its construction to demonstrate its generalizability in patients.

### Prospective

- 1. Promote the application of ANN in the MSD. Incorporating artificial neural networks into clinical settings can enable clinicians to predict disease progression and functional recovery faster and more accurately.
- 2. Optimize the quality of the data set. The model should be built by selecting samples with different etiology, disease duration, age, ethnicity, and sex, the number of layers and complexity of the algorithm model should be determined according to the amount of data to ensure that the trained ANN models have better clinical adaptability and benefit the clinical treatment.
- 3. Adjust legal regulations. The artificial intelligence technology represented by ANN requires a large amount of data input related to clinical parameters (e.g., images, and videos). Adequate laws related to the use of ANN in healthcare are necessary to ensure the protection of patient privacy, while reasonably allocating the responsibility in case of errors in artificial neural network models.

## Conclusion

This scoping review provides preliminary evidence that ANN can provide accurate prognosis prediction for MSDs by demographic information of patients and clinical characteristics of diseases. ANN models are superior to other traditional prediction models such as LR and deserve to be tested and replicated in other MSD types. The weaknesses highlighted must be addressed in future studies to enable ANNs models to better contribute to clinical decision making.

## Supplementary Information

**Additional file 1.**

## Data Availability

The datasets used and/or analysed during the current study are available from the corresponding author on reasonable request.
