# Supplementary material for: Use of artificial neural networks in the prognosis of musculoskeletal diseases—a scoping review
Source: BMC Musculoskelet Disord. 2023 Feb 1;24:86. doi: 10.1186/s12891-023-06195-2 (PMC9890715; doi:10.1186/s12891-023-06195-2)
Supplement: Supplementary file 1 — Additional file 1. [file 12891_2023_6195_MOESM1_ESM.docx]

Supplemental file: search strategy

Cochrane：

MeSH descriptor: [Neural Networks, Computer] explode all trees

MeSH descriptor: [Musculoskeletal Diseases] explode all trees

Result: 8


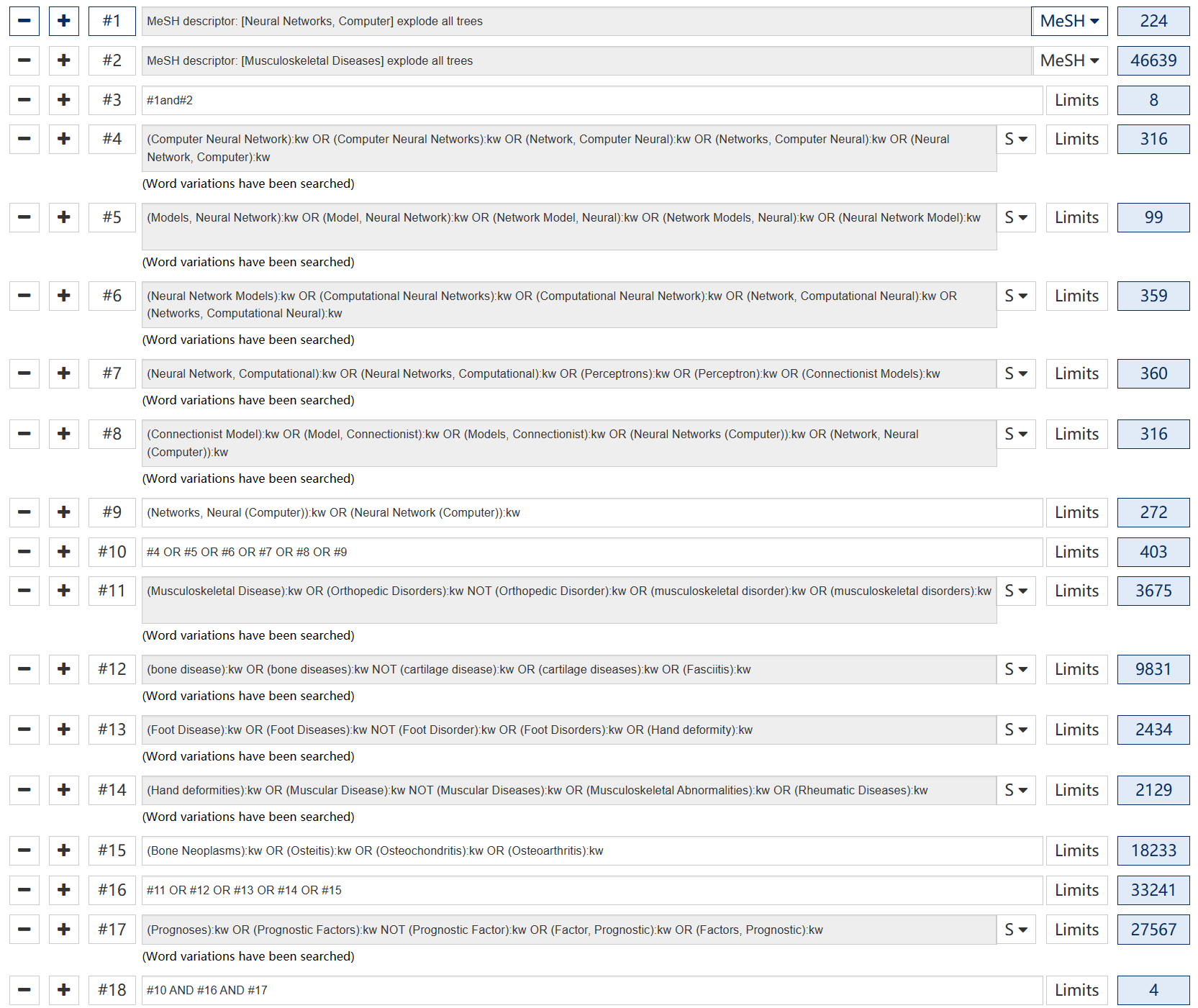


Keywords reseach: 4

**Total: 12**

**Embase：**

'artificial neural network'/syn

'musculoskeletal disease'/syn

'prognosis'/syn

**Total: 123**

**Pubmed:**

(((((((((((((((((((((((((((Neural Networks, Computer[MeSH Terms]) OR (Computer Neural Network)) OR (Computer Neural Networks)) OR (Network, Computer Neural)) OR (Networks, Computer Neural)) OR (Neural Network, Computer)) OR (Models, Neural Network)) OR (Model, Neural Network)) OR (Network Model, Neural)) OR (Network Models, Neural)) OR (Neural Network Model)) OR (Neural Network Models)) OR (Computational Neural Networks)) OR (Computational Neural Network)) OR (Network, Computational Neural)) OR (Networks, Computational Neural)) OR (Neural Network, Computational)) OR (Neural Networks, Computational)) OR (Perceptrons)) OR (Perceptron)) OR (Connectionist Models)) OR (Connectionist Model)) OR (Model, Connectionist)) OR (Models, Connectionist)) OR (Neural Networks (Computer))) OR (Network, Neural (Computer))) OR (Networks, Neural (Computer))) OR (Neural Network (Computer))

((((((((((((((((((((((((Musculoskeletal Diseases[MeSH Terms]) OR (Musculoskeletal Disease)) OR (Orthopedic Disorders)) OR (Orthopedic Disorder)) OR (musculoskeletal disorder)) OR (musculoskeletal disorders)) OR (bone disease)) OR (bone diseases)) OR (cartilage disease)) OR (cartilage diseases)) OR (Fasciitis)) OR (Foot Disease)) OR (Foot Diseases)) OR (Foot Disorder)) OR (Foot Disorders)) OR (Hand deformity)) OR (Hand deformities)) OR (Muscular Disease)) OR (Muscular Diseases)) OR (Musculoskeletal Abnormalities)) OR (Rheumatic Diseases)) OR (Bone Neoplasms)) OR (Osteitis)) OR (Osteochondritis)) OR (Osteoarthritis)

(((((Prognosis[MeSH Terms]) OR (Prognoses)) OR (Prognostic Factors)) OR (Prognostic Factor)) OR (Factor, Prognostic)) OR (Factors, Prognostic)

**Total: 115**

**Web of science:**

TS=(Neural Networks, Computer OR Computer Neural Network OR Computer Neural Networks OR Network, Computer Neural OR Networks, Computer Neural OR Neural Network, Computer OR Models, Neural Network OR Model, Neural Network OR Network Model, Neural OR Network Models, Neural OR Neural Network Model OR Neural Network Models OR Computational Neural Networks OR Computational Neural Network OR Network, Computational Neural OR Networks, Computational Neural OR Neural Network, Computational OR Neural Networks, Computational OR Perceptrons OR Perceptron OR Connectionist Models OR Connectionist Model OR Model, Connectionist OR Models, Connectionist OR Neural Networks (Computer) OR Network, Neural (Computer) OR Networks, Neural (Computer) OR Neural Network (Computer))

TS=(Musculoskeletal Diseases OR Musculoskeletal Disease OR Orthopedic Disorders OR Orthopedic Disorder OR musculoskeletal disorder OR bone disease OR cartilage disease OR Fasciitis OR Foot Disease OR Foot Disorder OR Hand deformity OR Muscular Disease OR Musculoskeletal Abnormalities OR Rheumatic Diseases OR Bone Neoplasms OR Osteitis OR Osteochondritis OR Osteoarthritis OR musculoskeletal disorders OR bone diseases OR cartilage diseases OR Fasciitis OR Foot Diseases OR Foot Disorders OR Hand deformities OR Muscular Diseases)

TS=( Prognosis OR Prognoses OR Prognostic Factors OR Prognostic Factor OR Factor, Prognostic OR Factors, Prognostic)

**Total: 40**
